# Phase II trial of sagopilone, a novel epothilone analog in metastatic melanoma

**DOI:** 10.1038/sj.bjc.6605931

**Published:** 2010-10-05

**Authors:** R C DeConti, A P Algazi, S Andrews, P Urbas, O Born, D Stoeckigt, L Floren, J Hwang, J Weber, V K Sondak, A I Daud

**Affiliations:** 1 Moffitt Cancer Center, 12902 Magnolia Drive, Tampa, FL, USA; 2 University of California, San Francisco, MTZ-A741, 1600 Divisadero Street, San Francisco, CA 94143, USA; 3 Drug Metabolism and Pharmacokinetics – Bioanalytics, Bayer Schering Pharma AG, Müllerstr, Berlin, Germany

**Keywords:** sagopilone, epothilones, melanoma, pharmacokinetics, toxicity, efficacy

## Abstract

**Background:**

Sagopilone is a novel fully synthetic epothilone with promising preclinical activity and a favourable toxicity profile in phase I testing.

**Methods:**

A phase II pharmacokinetic and efficacy trial was conducted in patients with metastatic melanoma. Patients had measurable disease, Eastern Cooperative Oncology Group performance status 0–2, adequate haematological, and organ function, with up to 2 previous chemotherapy and any previous immunotherapy regimens. Sagopilone, 16 mg m^−2^, was administered intravenously over 3 h every 21 days until progression or unacceptable toxicity.

**Results:**

Thirty-five patients were treated. Sagopilone showed multi-exponential kinetics with a mean terminal half-life of 64 h and a volume of distribution of 4361 l m^−2^ indicating extensive tissue/tubulin binding. Only grade 2 or lower toxicity was observed: these included sensory neuropathy (66%), leukopenia (46%), fatigue (34%), and neutropenia (31%). The objective response rate was 11.4% (one confirmed complete response, two confirmed partial responses, and one unconfirmed partial response). Stable disease for at least 12 weeks was seen in an additional eight patients (clinical benefit rate 36.4%).

**Conclusion:**

Sagopilone was well tolerated with mild haematological toxicity and sensory neuropathy. Unlike other epothilones, it shows activity against melanoma even in pretreated patients. Further clinical testing is warranted.

Melanoma is the most aggressive type of skin cancer; it is rapidly increasing in incidence worldwide. It is estimated that over 60 000 new cases and over 8000 deaths (Jemal *et al*, 2008) resulted from this disease in the United States in 2008. The overall prognosis of metastatic melanoma remains very poor (Barth *et al*, 1995) owing to the lack of effective, tolerable, non-cross-resistant therapeutic options. Current therapeutic options are poor and are not associated with a survival benefit (Atkins *et al*, 1999, 2000).

Taxane-based combinations are emerging as a treatment option in melanoma, despite significant limitations. Although phase II trials of paclitaxel have reported varying degrees of efficacy, a recent, large phase III trial of single-agent paclitaxel in front-line metastatic melanoma patients showed very low objective response rates (4.4%) and limited median overall survival (Einzig *et al*, 1991; Aamdal *et al*, 1994; Bedikian *et al*, 1995; Hauschild *et al*, 2009a, 2009b). However, in combination with carboplatin, responses have been seen in 11–36% (Hauschild *et al*, 2009a) of pretreated patients. Paclitaxel has several other drawbacks; owing to its insolubility, it is solubilised with Cremophor EL, a castor oil derivative that can induce potentially life-threatening hypersensitivity reactions (ten Tije *et al*, 2003). In addition, taxanes have poor penetration into the central nervous system (Glantz *et al*, 1995), limiting their utility in melanoma where CNS involvement is common, and they are susceptible to chemotherapy resistance mechanisms such as overexpression of the multidrug resistance-1 (MDR-1) transporter (Fojo and Menefee, 2007).

Epothilones are a newer class of microtubule-stabilising agents designed to overcome some of the limitations of taxanes. Although some epothilones, notably ixabepilone, have proven their efficacy in both taxane-sensitive and taxane-refractory solid tumours (Low *et al*, 2005; Perez *et al*, 2007; Rosenberg *et al*, 2007; Vansteenkiste *et al*, 2007; Burtness *et al*, 2008; Thomas, 2008), in melanoma, epothilones have so far failed to show significant activity. For example, patupilone showed no activity in one study (Daud, 2009), and ixabepilone, a second-generation epothilone showed no objective tumour responses, and significant treatment-related toxicities (Pavlick *et al*, 2004).

Preclinical data suggest that sagopilone (also called ZK-EPO), a fully-synthetic, third-generation epothilone may be more effective than other tubulin-active drugs in patients with advanced melanoma. Sagopilone evades the MDR-1 efflux pump, and, unlike the earlier generation epothilones, it is rapidly and efficiently taken up into tumour cells (Klar *et al*, 2006; Hoffmann *et al*, 2008). In a phase I clinical trial in patients with advanced solid tumours, antitumour activity was observed in 15 of 44 patients, including at least one patient with ocular melanoma (Schmid *et al*, 2010). Phase II trials have shown that sagopilone is active in ovarian cancer (Rustin *et al*, 2007; McMeekin *et al*, 2008), small-cell lung cancer (Gatzemeier *et al*, 2007; Fischer *et al*, 2008), and androgen-independent prostate cancer (Beer *et al*, 2008; Graff *et al*, 2008). Unlike taxanes and some epothilones, sagopilone crosses the blood–brain barrier, and antitumour efficacy has been shown in a xenograft model of melanoma brain metastases (Hoffmann *et al*, 2009) and in brain metastases from small-cell lung cancer (Christoph *et al*, 2009).

Sagopilone also has a more favourable toxicity profile than other tubulin-active drugs. Sagopilone is more water soluble than earlier epothilones, and formulation with Cremophor EL is not required (Klar *et al*, 2006; Hoffmann *et al*, 2008). The most common toxicities associated with sagopilone at the recommended phase II dose of 16 mg m^−2^ every 21 days have included grade 1–2 peripheral sensory neuropathy, fatigue, nausea, and, less commonly, mild haematological toxicity (Rustin *et al*, 2007).

We conducted a phase II trial of sagopilone in patients with metastatic melanoma to determine whether it has the potential to expand the therapeutic arsenal of non-cross-resistant chemotherapeutics in metastatic melanoma.

## Methods

### Study design

This was a prospective, single arm, phase II trial designed to determine the magnitude of benefit and safety profile of sagopilone in melanoma. In addition, the first 10 patients underwent pharmacokinetic testing and evaluation. The trial was approved by the Scientific Review Committee and the Institutional Review Board at the Moffitt Cancer Center. Accrual began in May 2007 and was completed in October 2008.

### Patients

Eligible patients had to have unresectable stage III or stage IV melanoma with measurable disease by the Response Evaluation Criteria in Solid Tumours (RECIST) (Therasse *et al*, 2000). Treatment with up to two previous chemotherapy regimens was allowed as long as there had been no previous treatment with tubulin-active drugs that are similar in mechanism of action to sagopilone, such as other epothilones, taxanes, or vinca alkaloids. Patients had to be ⩾18 years of age with an Eastern Cooperative Oncology Group performance status of 0–2, adequate bone marrow function (white blood cell count >3.0 × 10^9^ l^−1^, haemoglobin >10 g dl^−1^, platelets >10^9^ l^−1^), adequate hepatic function (total bilirubin <1.5 times the upper limit of normal, and AST and ALT <5 times the upper limit of normal), and adequate renal function (creatinine less than 2 mg dl^−1^). Patients were excluded if they had New York Heart Association class III or IV congestive heart failure, unstable angina pectoris, or cardiac arrhythmias requiring continuous treatment. Patients with grade 2 or higher sensory neuropathy of any aetiology were also excluded due concern that sagopilone could exacerbate their symptoms. Patients with CNS involvement were eligible if they were asymptomatic, and if the lesions were radiographically stable over a period of at least 8 weeks.

### Study treatment

Patients were treated with 16 mg m^−2^ of sagopilone administered as a single 3-h intravenous infusion every 21 days. Before each treatment, patients were premedicated with either granisetron or ondansetron and additional antiemetics if needed. Chemotherapy was continued until disease progression, withdrawal of consent, or for unacceptable treatment-associated toxicity. Treatment was discontinued if a patient incurred grade 4 neurotoxicity or grade 2 or higher neurotoxicity lasting longer than 5 weeks with or without treatment delays. Any other grade 3 or 4 toxicity that persisted despite maximal supportive care led to dose reduction, treatment interruption, or discontinuation of sagopilone therapy at the discretion of the investigators.

### Bioanalysis

Plasma levels of sagopilone were determined by liquid chromatography/mass spectrometry (LC/MS/MS) (US Food and Drug Administration, 2001) in the first 10 patients on the study. Samples were obtained at the following time points: before the initiation of sagopilone; at 30, 175, 190, and 210 min; at 5, 8, and 27 h; and at 1 week after the initiation of the infusion. In all cases, 1 ml blood samples were collected in lithium heparin tubes, stabilised by Pefabloc, and centrifuged at 4000 r.p.m. for 10 min within 30 min after collection. From the supernatant, two aliquots of 250 *μ*l were transferred into test tubes and stored at −80°C until analysis. The quantitative analysis of sagopilone plasma levels was performed at the Function Bioanalytics Laboratory, Bayer Schering Pharma AG Berlin, Germany. In a first step, sagopilone was isolated from the plasma by liquid–liquid extraction, followed by the LC/MS/MS analysis. The lower limit of quantification (LLOQ) for the determination of sagopilone concentrations in human Li-heparin plasma containing Pefabloc was established to be 100 pg ml^−1^ during method validation. In this study, accuracy and precision was ensured by means of quality control samples. Over the calibrated range (0.100–100 ng ml^−1^), the mean accuracy varied from 98.0 to 111% and the precision from 4.4 to 8.7%.

### Pharmacokinetics

Pharmacokinetic parameters were calculated using the standard software (EPSKinetica, version 2.6.1, Thermo Fisher Scientific, Waltham, MA, USA) without recourse to model assumptions. The pharmacokinetic evaluation was based on individual plasma concentration–time values of sagopilone using actual blood sampling times. *C*_max_ and *T*_max_ were directly read off the concentration–time profiles. The area under the curve (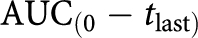 and AUC) was calculated according to the mixed log–linear trapezoidal rule. All plasma concentration values below the LLOQ were set to zero. The terminal disposition rate constant (*λz*) was calculated by means of regression analysis of the perceivable linear part of the curve in a semilogarithmic plot (*λz*: slope of the regression line). The corresponding terminal half-life (*t*_1/2_) was calculated by: *t*_1/2_=ln2/*λz*. Individual values were not accepted if the time range covered by the perceivable linear part of the curve was less than two half-lives. In all cases, at least three data points were used for the half-life calculation of each disposition phase. The AUC was calculated according to the following equation: AUC=
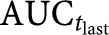
+(computed *C*_last_/*λz*), with computed *C*_last_ being the concentration calculated for the time point (*t*_last_) with the last quantifiable concentration, and *λz* being the terminal disposition rate constant.

### Response assessment

The primary end point in this study was assessment of objective tumour response by the RECIST criteria. Secondary end points included time to progression, overall survival, and tolerability. Tumour assessments using the modified RECIST criteria were made at baseline and at the end of every second cycle (i.e. every 6 weeks). Partial and complete responses were defined by the best treatment response achieved. Stable disease was defined as maintenance between a 30% reduction and a 20% increase of tumour size over 12 weeks or longer. Stable disease was measured from the start of the treatment until the criteria for disease progression were met, and formal radiographic confirmation was not required in accordance with the RECIST criteria. Patients were assessed for adverse events based on clinical and laboratory data on treatment days 1, 8, and 15 of the first 21-day cycle, and then every 3 weeks on day 1 of each subsequent cycle.

### Statistics

A Gehan–Simon optimal two-stage trial design was used to limit patient accrual in case the trial drug proved ineffective. Accrual of 33 evaluable patients was planned; if at least 1 of the first 15 patients had an objective response as defined by the RECIST criteria, accrual would continue. The ‘null’ hypothesis assumed a response probability of 1% (spontaneous remission), whereas the target response rate was 15%. The alpha level of the design was 0.03 and the power was 90%. Descriptive Kaplan–Meier survival analysis was performed and subgroup survival comparisons using log-rank were performed using SPSS version 17 (Chicago, IL, USA). Adverse events were graded using the Common Terminology Criteria for Adverse Events version 3.0.

## Results

### Patients

Thirty-nine patients were consented and screened for this trial. Four were ineligible based on the screening criteria and were not treated with sagopilone. Thirty-five patients, all with stage IV melanoma, were treated with at least one dose of sagopilone and included in response and toxicity assessments. Patient demographics, staging information, and previous treatment histories are summarised in Table 1. The majority of patients had stage IVC disease (74.3%) and lactate dehydrogenase (LDH) elevations were seen in nearly half of patients (45.7%). Most patients (65.7%) had been treated previously with cytotoxic chemotherapy (including two treated regionally with isolated limb perfusion) and a substantial proportion had received immunotherapy (28.6%). Twenty-one patients (60%) received dacarbazine or temozolomide. Two patients were previously exposed to cisplatin. The average number of chemotherapy cycles received was three or fewer for each regimen, except for the combination of karenitecin with valproic acid. Two patients were treated with this combination received an average of 10 cycles. The median age of the patients treated in this study was 68.7 (±1.7 years).

### Pharmacokinetics

**Figure 1:**
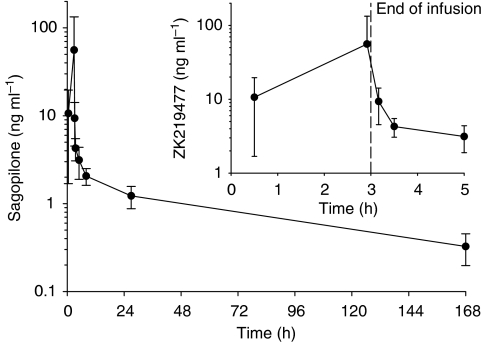
Mean plasma concentration–time curve of sagopilone after single intravenous infusion of 16 mg m^−2^ sagopilone over 3 h (*N*=9–10).

Nine of the subjects were evaluable since one patient was excluded from the PK analysis owing to three missing PK samples necessary to define the *C*_max_ and AUC. Maximum average sagopilone concentrations of 33.3 ng ml^−1^ were reached 2.92 h after the start of the infusion (Figure 1). Plasma concentrations dropped about 10-fold within 30 min after the end of infusion. Thereafter, the concentration–time profile turned into a slow terminal disposition phase. Drug disposition appeared to be multi-exponential, with sagopilone plasma concentrations decreasing to about 10% of peak concentration by 0.5 h after the end of the 3-h intravenous infusion. After the rapid distribution phase, a long terminal disposition phase followed (mean terminal half-life 64.1 h). The large apparent volume of distribution indicates extensive tissue/tubulin binding. Therefore, the long terminal half-life was thought to reflect the release of sagopilone from deep tissue compartments rather than the actual rate of its metabolism or excretion. The rate of total body clearance was 120 l h^−1^ (2000 ml min^−1^) and the apparent volume of distribution during the terminal phase was 4361 l m^−2^. The net average area under the plasma concentration–time curve was 252 ng.h ml^−1^. The kinetic parameter estimates observed in this study were similar to those determined in previous studies at the same dose and dosing schedule.

### Toxicity and adverse events

Chemotherapy was generally well tolerated and no grade 3 or higher adverse events were attributable to treatment with sagopilone. The most common adverse events included grade 1 and 2 haematological toxicity (40% of patients), and grade 1 and 2 motor (22.9%) and sensory neuropathy (51.4%). Of 12 patients receiving more than two cycles of sagopilone, five (43%) experienced grade 1 or 2 motor neuropathy and seven (58%) experienced grade 1 or 2 sensory neuropathy. One patient who received a total of 10 cycles of sagopilone required a one-time treatment interruption for grade 2 motor neuropathy, and a second patient who received a total of 20 cycles required a dose reduction owing to grade 2 sensory neuropathy. Two patients died before the first restaging scans. Both cases are reported in this manuscript as treatment failures, although neither death was thought related to treatment-associated toxicity: one patient opted to enter hospice before follow-up imaging and the other had sudden death not thought linked to treatment. Table 2 summarises adverse events that were judged to be possibly, probably, or definitely linked to sagopilone exposure.

### Response

**Figure 2:**
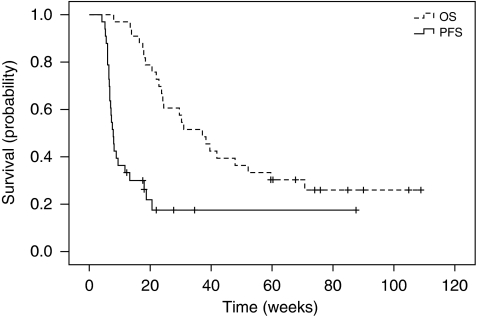
Progression-free survival (PFS) and overall survival (OS) in 33 evaluable patients.

One patient had 20 cycles of sagopilone, had a complete response, and has remained without evidence of disease progression (last scan, February 2010), and two other patients had confirmed partial responses. One patient had an unconfirmed partial response with 48% tumour reduction by the RECIST criteria, but after the next chemotherapy cycle, he had bleeding from a bladder metastasis detected on cystoscopy that was thought to pre-date treatment on study. The pre-existing nature of the lesion could not be confirmed; however, since the patient did not undergo pretreatment cystoscopy. Including this patient, the overall objective response rate was 11.4%. In addition, eight patients had stable disease for an interval of 12 weeks or longer, such that 12 of 35 patients (34.3%) derived clinical benefit from sagopilone (Table 3). Eight of 12 patients deriving clinical benefit from sagopilone had been treated with another chemotherapy regimen previously. Only the 12 patients deriving clinical benefit from sagopilone received more than two cycles of treatment and the median number of sagopilone cycles received by patients benefiting from treatment was 6. The median PFS was 7.5 weeks (95% confidence interval (CI): 6.4–8.7 weeks) and the median OS was 31.0 weeks (95% CI: 14.9–47.1 weeks; Figure 2).

### Subgroup analysis

**Figure 3:**
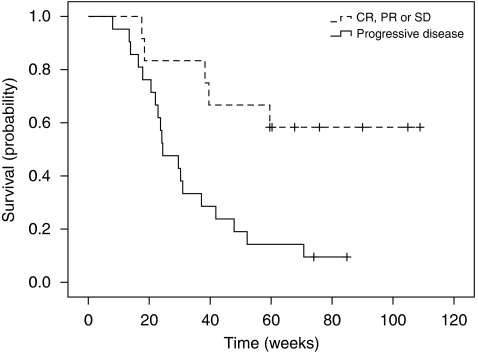
Overall survival in patients with progressive disease *vs* patients with stable disease (*N*=21), partial treatment response, or complete treatment response as the best response to sagopilone (*N*=12).

Patients showing a clinical benefit (combined complete response+partial response+stable disease) had a median OS not reached at a median follow-up of 59.9 weeks, in comparison with patients with progressive disease who had a median overall survival of 24.4 weeks (*P*<0.01; Figure 3). Patients with LDH less than the upper limit of normal had a longer survival than those with an elevated LDH (median OS 55.9 weeks *vs* median OS of 20.6 weeks, *P*<0.001). No difference was seen for OS between patients ⩾60 compared with patients younger than 60, between men and women, or between patients with stage IVA, IVB, and IVC disease.

## Discussion

Treatment options in metastatic melanoma have been limited by the lack of non-cross-resistant treatment options. As most effective chemotherapy agents in melanoma are myelosuppressive, it is difficult to combine them without significant dose reduction. Sagopilone notably lacks haematological toxicity. Data from this study suggest that sagopilone may offer a new therapeutic option that shows evidence of effectiveness and may be a good candidate for combination regimens. Objective tumour responses were seen in 11.4% of treated patients and 34.3% of patients had a potential clinical benefit from sagopilone. The 12-month overall survival in this study, 34.3%, also compares favourably to the benchmark overall survival of 25.5% (95% CI: 23.6–27.4%) established in a recent meta-analysis of over 2000 advanced melanoma patients enrolled in phase II clinical trials (Korn *et al*, 2008).

Sagopilone appears to be more active than other tubulin-active agents. Paclitaxel has been extensively studied in melanoma and objective response rates in the first-line setting have ranged from 0 to 16% in small phase II trials (Einzig *et al*, 1991; Aamdal *et al*, 1994; Bedikian *et al*, 1995). The largest data set of paclitaxel-treated melanoma patients using RECIST measurements is the SYMMETRY Phase III trial in which paclitaxel monotherapy was compared to paclitaxel with esclomolol. In this trial, objective responses to paclitaxel were seen in only 4.4% of patients treated. Paclitaxel is much less soluble than sagopilone and needs to be formulated with Cremaphor EL, which can induce serious hypersensitivity reactions in 11–18% of patients even with high-dose steroid premedication (Einzig *et al*, 1991; Bedikian *et al*, 1995). Sagopilone, which does not require formulation with Cremaphor EL, did not induce any signs or symptoms of hypersensitivity reactions in this study even without steroid pretreatment. Also in contrast to paclitaxel, grade 3 and 4 haematological toxicity was not observed, and no patient developed febrile neutropenia. Although sagopilone penetrates the CNS, neurological toxicity consisted primarily of mild-to-moderate sensory neuropathy. Sagopilone also appears more active than earlier generation epothilones that were also associated with higher degrees of toxicity and little evidence of clinical benefit to melanoma patients. Both patupilone and ixabepilone have been tested in phase II trials and have shown no objective responses or notable disease stabilisation (Pavlick *et al*, 2004; Daud, 2009). Ixabepilone has significant haematological toxicity in addition to neurotoxicity (Pavlick *et al*, 2004) and patupilone is associated with GI toxicity, especially diarrhoea (Daud, 2009; Hussain *et al*, 2009).

The distinctive clinical profile of sagopilone may be, in part, due to its unique pharmacokinetic and pharmacodynamic profile. In *in vitro* assays, sagopilone localises almost exclusively to the cytoskeletal compartment of melanoma cells, associates strongly with microtubules, and induces tubulin polymerisation at a rate that is approximately 50% higher than the rate observed in melanoma cells treated with patupilone (Hoffmann *et al*, 2008). More importantly, unlike some earlier generation epothilones, sagopilone is rapidly and efficiently taken up into tumour cells, and because it evades the MDR-1 efflux pump P-glycoprotein, it is maintained within these cells (Klar *et al*, 2006; Hoffmann *et al*, 2008). Sagopilone biodistribution has previously been described as multi-compartmental, with a high volume of distribution and high clearance that likely indicate uptake into tissues and a slow release (Schmid *et al*, 2010). In this study, with a larger cohort of patients, we report AUC (252 *vs* 602 ng.h ml^−1^) and *C*_max_ measurements (33.3 *vs* 101 ng.h ml^−1^) lower than that observed previously. Notably, the values previously reported by Schmid *et al* (2010) were derived from a single patient and much more extensive prior treatment was the norm in that study.

The majority of patients benefiting from sagopilone in this study had been pretreated, suggesting that sagopilone may not be fully cross-resistant with dacarbazine and/or platinum compounds. The incidence of neurological toxicity was substantially lower than that reported in a recent phase I trial (Schmid *et al*, 2010), perhaps because patients in the previous study were more heavily pretreated (median of previous regimens 3 *vs* 1) and more likely to have had received previous neurotoxic agents, including taxanes, vinca alkyloids, and platinum compounds. In addition, sagopilone was not associated with significant haematological toxicity in this study, making it a better choice for combination therapy. These properties may be significant as the combination of carboplatin and paclitaxel appears to be active in melanoma (Hauschild *et al*, 2009a, 2009b) and is emerging as a standard therapy for metastatic melanoma. This regimen has several limitations; these include its limited efficacy and cumulative haematological toxicity. Sagopilone, with its activity and toxicity profile, is a viable candidate for combination therapy.

## Figures and Tables

**Table 1:** Patient demographics, stage, and previous treatment

**Factor**	**Category**		***N***	**%**
Gender	Male		24	68.6
	Female		11	31.4
Stage	IV – M1a		1	2.9
	IV – M1b		8	22.9
	IV – M1c		26	74.3
Previous treatments	Surgery		29	82.9
	Radiation		7	20.0
	Chemotherapy	Actinomycin D	1	2.9
		Carmustine	1	2.9
		Cisplatin	2	5.7
		DTIC/temozolomide	21	60.0
		Karenitecin	2	5.7
		Melphalan ILP	2	5.7
	Immunotherapy	Interferon	9	25.7
		IL-2	2	5.7
		Ipilimumab	1	2.9
		Intralesional GM-CSF	1	2.9
		IL-12 electroporation	1	2.9
	Kinase inhibitors	Sunitinib	1	2.9
		Dasatinib	1	2.9
		ATN 224-007	1	2.9
	HDAC inhibitor	Valproic acid	2	5.7
	Patients treated previously	Adjuvant/regional	14	40.0
		For systemic disease	21	60.0
Baseline LDH	Normal		19	54.3
	Elevated		16	45.7
ECOG PS	0		24	68.6
	1		10	28.6
	2		1	2.9
Primary Site	Sun-damaged skin		3	8.6
	Non-sun-damaged skin		13	37.1
	Acral		4	11.4
	Ocular		7	20.0
	Mucosal		1	2.9
	Unknown		7	20.0

Abbreviations: HDAC=histone deacetylase; LDH=lactate dehydrogenase; DTIC=dacarbazine; ECOG=Eastern Cooperative Oncology Group; PS=performance status; ILP=isolated limb perfusion; GM-CSF=granulocyte–macrophage colony-stimulating factor; IL-2=interleukin-2; IL-12=interleukin-12.

Most patients received more than one previous treatment. Some systemic agents were given in combination.

**Table 2:** Percentage of 35 patients treated with sagopilone experiencing adverse events by category

		**Grade (%)**		
**Group**	**Toxicity**	**1**	**2**	**Total (%)**
Haematological	Anaemia	8.6	8.6	17.2
	Neutropenia	5.7	5.7	11.4
	Thrombocytopenia	5.7	8.6	14.3
Cardiac	Tachycardia	2.9	—	2.9
	Pericardial effusion	2.9	—	2.9
	Hypertension	—	2.9	2.9
Constitutional	Fatigue	25.7	5.7	31.4
	Fever	5.7	—	5.7
Dermatological	Alopecia	11.4	—	11.4
	Rash	8.6	—	8.6
	Dry skin	2.9	—	2.9
Gastrointestinal	Anorexia	14.3	—	14.3
	Nausea	31.4	—	31.4
	Other	14.3	—	14.3
Laboratory	Hepatic	5.7	2.9	8.6
	Other	5.7	—	5.7
Neurological	Motor neuropathy	8.6	14.3	22.9
	Sensory neuropathy	45.7	5.7	51.4
	Other	2.9	2.9	5.8
Pain		40.0	2.9	42.9
Respiratory	Cough	5.7	—	5.7
	Dyspnoea	2.9	—	2.9

Only the highest-grade event was included for each patient in each category. No grade 3 or higher adverse events were observed.

**Table 3:** Best treatment response by the RECIST criteria

**Best treatment response**	***N***	**%**
Complete response	1	2.9
		
*Partial response*		
Confirmed	2	5.7
Unconfirmed	1	2.9
		
Stable disease	8	22.9
Progressive disease	23	65.7
Total	35	100

Abbreviation: RECIST=Response Evaluation Criteria in Solid Tumours.
